# The Expression of NTAL and Its Protein Interactors Is Associated With Clinical Outcomes in Acute Myeloid Leukemia

**DOI:** 10.1016/j.mcpro.2021.100091

**Published:** 2021-05-07

**Authors:** Carolina Hassibe Thomé, Germano Aguiar Ferreira, Diego Antonio Pereira-Martins, Guilherme Augusto dos Santos, Douglas R. Almeida-Silveira, Isabel Weinhäuser, Gustavo Antônio de Souza, Roos Houtsma, Jan Jacob Schuringa, Eduardo M. Rego, Vitor M. Faça

**Affiliations:** 1 Department of Biochemistry and Immunology, Ribeirão Preto Medical School, University of São Paulo, Ribeirão Preto, Brazil; 2 Department of Internal Medicine, Ribeirão Preto Medical School and Center for Cell Based Therapy, University of São Paulo, Ribeirão Preto, Brazil; 3 Department of Hematology, Cancer Research Centre Groningen, University Medical Centre Groningen, University of Groningen, Groningen, the Netherlands; 4 Hematology Division, LIM31, Medical School of University of São Paulo, São Paulo, Brazil; 5 Department of Hematology, AC Camargo Cancer Center, São Paulo, Brazil; 6 Department of Immunology, Oslo University Hospital - Rikshospitalet, Oslo, Norway

**Keywords:** NTAL, acute myeloid leukemia, interactors, AML, acute myeloid leukemia, APL, acute promyelocytic leukemia, ATRA, all-*trans* retinoic acid, CSF, colony-stimulating factor, CT, control, DMEM, Dulbecco's modified Eagle's medium, DRM, detergent-resistant membrane, FDR, false discovery rate, GMP, granulocyte–macrophage progenitor, GO, Gene Ontology, GSEA, gene set enrichment analysis, HCD, high-energy collision dissociation, hr, human recombinant, HSCs, hematopoietic stem cells, iBAQ, intensity-based absolute quantification, IP, immunoprecipitation, KD, knockdown, LAT2, linker for activation of T cells 2, MAPK, mitogen-activated protein kinase, mTOR, mammalian target of rapamycin, NTAL, non–T cell activation linker, OS, overall survival, PLA, proximity ligation assay

## Abstract

Non–T cell activation linker (NTAL) membrane protein depletion from lipid rafts by alkylphospholipids or downregulation by shRNA knockdown decreases cell viability through regulation of the Akt/PI3K pathway in mantle cell lymphoma and acute promyelocytic leukemia cells. Here, we confirmed that the knockdown of NTAL in acute myeloid leukemia (AML) cell lines was associated with decreased cell proliferation and survival. Similarly, a xenograft model using AML cells transduced with NTAL–shRNA and transplanted into immunodeficient mice led to a 1.8-fold decrease in tumor burden. Using immunoprecipitation, LC–MS/MS analysis, and label-free protein quantification, we identified interactors of NTAL in two AML cell lines. By evaluating the gene expression signatures of the NTAL protein interactors using the PREdiction of Clinical Outcomes from Genomic Profiles database, we found that 12 NTAL interactors could predict overall survival in AML, in at least two independent cohorts. In addition, patients with AML exhibiting a high expression of NTAL and its interactors were associated with a leukemic granulocyte–macrophage progenitor–like state. Taken together, our data provide evidence that NTAL and its protein interactors are relevant to AML cell proliferation and survival and represent potential therapeutic targets for granulocyte–macrophage progenitor–like leukemias.

## Highlights

• NTAL knockdown in AML cells decreased cell proliferation and survival.
• We discovered 49 NTAL interactors presents in AML cells.
• Patients with *de novo* AML displayed a high expression of NTAL and its interactors.
• NTAL and its interactors were associated with overall survival in patients with AML.

## Graphical Abstract

**Figure:**
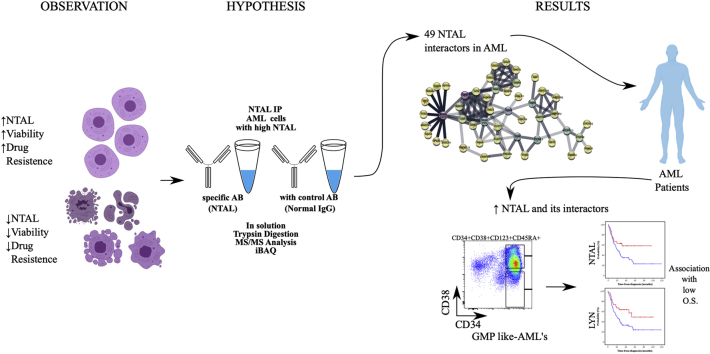

## In Brief

Here, we demonstrated that the knockdown of non–T cell activation linker (NTAL) in acute myeloid leukemia (AML) cells was linked to reduced cell proliferation and survival *in vitro* and *in vivo*. In addition, we identified NTAL interactors in AML using label-free protein quantification. NTAL interactors presented a high expression in patients with AML, being associated with a leukemic granulocyte–macrophage progenitor-like state. Finally, NTAL interactors were capable to predict survival in a large subset of patients with AML. These data provide evidence that NTAL and its interactors could represent potential therapeutic targets for granulocyte–macrophage progenitor-like leukemias.

The lipid raft adaptor membrane protein non–T cell activation linker (NTAL), or linker for activation of T cells 2 (LAT2), was first described as a functional homolog of LAT, an adaptor for the T-cell receptor signaling hub (1, 2, 3). NTAL is expressed by different types of cells, including acute myeloid leukemia (AML) blasts, and absent in T cells (4). NTAL possesses tyrosine-based activation motifs and docking sites for proteins as Grb2, Sos1, Gab1, and c-Cbl (5, 6, 7), which indicates that NTAL can have multiple interacting partners to activate different downstream signaling cascades.

We have previously shown that the depletion of NTAL from lipid rafts upon the treatment by alkylphospholipids was associated with impairment of Akt activation in an acute promyelocytic leukemia (APL) model (8). Also, the depletion of NTAL by shRNA knockdown (KD) leads to increased sensitivity to the proapoptotic activity of arsenic trioxide and decreased cell proliferation (8). Recently, we extended the analysis about the functional role of NTAL in APL, showing that reduced NTAL levels were associated with increased all-*trans* retinoic acid (ATRA)–induced cell differentiation, reactive oxygen species generation, Ras activation, and Akt/mammalian target of rapamycin (mTOR) pathway inhibition. Furthermore, a retrospective analysis of NTAL expression in a cohort of patients with APL treated with ATRA plus anthracycline-based chemotherapy revealed that high NTAL expression was associated with high leukocyte counts and decreased overall survival (OS) (9).

Although NTAL appears to impact the Akt signaling pathway in leukemia (8), the exact mechanisms of action and interactors are still unknown in leukemic cells. Of note, Akt pathway is constitutively activated in human leukemia cells, and therapeutic approaches that exploit Akt as a target represent an important field of investigation (10). Therefore, the study of NTAL interaction partners and signaling pathways could provide new insights into the leukemic molecular mechanism and eventually uncover new therapeutic opportunities.

In the present study, we highlight that NTAL is highly expressed in hematologic and lymphoid neoplastic cell lines. KD of NTAL in AML cell lines reduced cell proliferation, survival, and tumor burden in an U937-xenograft model, reinforcing the hypothesis that NTAL contributes to tumor growth. In addition, combining immunoprecipitation (IP) and LC–MS/MS–based protein identification and quantification, we describe and validate new NTAL interactors. By bioinformatic interrogation of NTAL interactors and their respective gene expression signatures, we demonstrate that several proteins, which are part of the NTAL network, also predict OS in AML, in at least two independent cohorts. Dichotomization of patients with AML into two groups (high *versus* low expression) associated patients with a high expression of NTAL interactors with an increased granulocyte–macrophage progenitor (GMP)–like leukemic signatures. In addition, 29 of 49 (59%) of the interactors are differentially expressed in AML samples in comparison with healthy hematopoietic stem cells (HSCs), establishing the relevance of NTAL and its network of protein interactions in AML biology and patient outcome.

## Experimental Procedures

### Cell Lines and Culture Procedures

The human AML cell lines, U937 (CRL-1593.2), THP1 (TIB-202), HL-60 (CCL-240), and K562 (CCL-243), were obtained from the American Type Culture Collection cell bank; the MV4-11 (ACC-102), Kasumi-1 (ACC-220), and OCI-AML3 (ACC-582) were obtained from the DSMZ cell bank; and 293FT cell lines were purchased from Invitrogen (Thermo Scientific). NB4 was kindly provided by Dr Pier Paolo Pandolfi (Harvard Medical School). Cells were cultured in RPMI1640 or Dulbecco's modified Eagle's medium (DMEM) or alpha-minimum essential medium or Iscove's modified Dulbecco's medium (Gibco), both supplemented with 10% or 20% (v/v) fetal bovine serum, 100 U/ml penicillin, and 100 μg/ml streptomycin (Gibco) at 37 °C with 5% CO_2_, accordingly with the cell line bank recommendation. Cell viability was determined by the trypan blue assay. Only cultures with more than 95% viability were used. *Mycoplasma* contamination was routinely tested (once per month). Only mycoplasma-free cells were used in the experiments. All cell lines were tested by short tandem repeat analysis (last evaluation—February 2020).

### Lentiviral Production

Lentiviral particles were produced in 293FT cells using the ViraPower Lentiviral Expression System (Life Technologies). Briefly, 1 μg of the plasmid of interest and 3 μg of packing plasmids: pLP1, pLP2, and pLP/VSVG, mixed in 12 μl of Lipofectamine 2000 in 2 ml of antibiotics-free and fetal bovine serum–free DMEM was added to 293FT cells overnight. The medium was then replaced with complete DMEM. After 48 h of transfection, the collected medium was centrifuged at 5000*g* for 15 min at 4 °C. Aliquots of lentiviral particles were immediately used for transfection or stored at −80 °C.

### Cell Transfection

Lentiviral particles containing MISSION TurboGFP Control Transduction Particles (pLKO.1) or MISSION shRNA Plasmid DNA NTAL (SHCLNV-NM_014146) (Sigma) were used for cell transfection. The shRNA sequence selection was performed using the Broad Institute RNAi consortium data bank (11). For transfection, 6.5 × 10^5^ U937 cells were plated with medium containing viral particles and 8 μg/ml polybrene (Sigma) overnight. Cells were washed twice and resuspended in complete medium. After 48 to 72 h, cells were selected with puromycin (0.5 μg/ml) (Sigma) for three to five passages (~10 days). U937 cells transduced with the sequence TNRC000129029 (KD—cells transduced with shRNA–NTAL) exhibited a higher level of NTAL inhibition compared with the control (CT—cells transduced with scrambled RNA) and were chosen for further functional assays (supplemental Fig. S1).

### Protein Extraction and Quantification

Cells were washed with PBS, lysed with lysis buffer (20 mM Tris–HCl [pH 7.5], 150 mM NaCl, 1 mM Na_2_EDTA, 1 mM EGTA, 1% Triton, 2.5 mM sodium pyrophosphate, 1 mM β-glycerophosphate, 1 mM Na_3_VO_4_, and 1 μg/ml leupeptin) and homogenized in a D-130 tissue homogenizer (Biosystems) at 15,000 rpm for 2 min on ice and centrifuged at 20,000*g* for 30 min at 4 °C. The protein concentration was determined by the Bradford method (Bio-Rad).

### Western Blotting

Cell lysates or eluted IP proteins were separated by SDS-PAGE and electrotransferred to polyvinylidene fluoride membranes (GE Lifesciences). Membranes were blocked with 5% nonfat dry milk in 0.1% Tween–Tris-buffered saline and incubated with a primary antibody following the manufacturer's instructions. The antibodies are listed in supplemental Table S1. The following secondary antibodies were used: horseradish peroxidase–conjugated goat anti-rabbit IgG (#7074) or goat anti-mouse IgG (#7076) secondary antibody or streptavidin–horseradish peroxidase conjugate (#3999) (Cell Signaling). Membranes were developed using ECL Western blotting Detection Reagents (GE Lifesciences). Images were acquired using a charged-coupled device camera (Image Quant LAS 4000 mini). Densitometric analysis was performed using the ImageJ software (imagej.nih.gov) (12), and bands were normalized to constitutive proteins. The values are presented as the NTAL–KD/CT ratio.

### Effect of NTAL on Akt Activation

U937 cells (CT and NTAL–KD) were maintained in culture in serum-free medium overnight (16 h). Cells were then stimulated with physiological doses of myeloid growth factors (10 ng/ml of human recombinant [hr]-IL-3 or 10 ng/ml of hr-granulocyte–macrophage colony-stimulating factor [CSF] or 10 ng/ml of hr-granulocyte CSF) (PeproTech). Aliquots were removed after 15 min of stimulation and assayed by antibody PathScan Intracellular Signaling Array Kit.

### PathScan Intracellular Signaling Array Kit

The PathScan Intracellular Signaling Array Kit containing fixed antibodies against phosphorylated proteins by the chemiluminescent sandwich ELISA format was used according to manufacturer's instructions (#7323; Cell Signaling). Images were analyzed with Image Studio Lite quantification software (version 4.0) (LI-COR), by loading the image as a grayscale picture. Each protein array dot was selected manually, and an average intensity was calculated for each protein. Normalization within one stimulation experiment was done by subtracting the intensity of the negative control dot from each value. For comparison of different conditions, sets were normalized so that the positive controls had nearly equal intensities.

### Murine Xenograft Model

Male 12-week-old NOD scid gamma mouse (*NOD.Cg-Prkdc*^*scid*^*Il2rg*^*tm1Wjl*^*/SzJ*) mice were maintained in our animal facility, receiving NUVITAL (autoclavable rodent pellets) and water (autoclaved) ad libitum, under a 12/12 light/dark cycle, at an environmental temperature of 23 °C and relative humidity of 55%. They were injected subcutaneously with 1 × 10^6^ CT cells into the right lateral flank and with the same number of NTAL–KD cells (U937 cells) into the left lateral flank. After 2 weeks (or if the tumors reached 1.5 cm of size), all the animals were euthanized and the tumors excised, weighed, and processed for further analysis. No randomization or exclusion criteria were used for animal studies. Animal procedures complied with the guidelines on animal experimentation for the protection and humane use of laboratory animals. The Ethics Committee for Animal Experimentation of The Faculdade de Medicina de Ribeirão Preto—USP approved the procedures used (protocol 135/2014).

### Histology and Immunohistochemistry

Fragments of each tumor were fixed in 10% formalin and embedded in paraffin. Hematoxylin and eosin staining and immunohistochemistry analysis against NTAL were performed on formalin-fixed and paraffin-embedded tissue sections (13).

### IP of NTAL and Its Interactors and Sample Processing for Proteomic Analysis

IP of proteins from the NB4 and U937 cell lysates was carried out in triplicate using protein extracts from 5 × 10^7^ cells. NTAL rabbit antibody (catalog no. 9533; Cell Signaling) or a rabbit anti-IgG antibody (catalog no. 3900; Cell Signaling) (0.014 μg) were incubated with 500 μg of each cell lysate overnight at 4 °C under constant agitation. Protein A/G magnetic beads (Pierce Biotechnology) were then incubated with lysate + plus antibody in cell lysis buffer for 1 h. Beads were washed with wash buffer (25 mM Tris–HCl, pH 7.5, 0.5 M NaCl, and 0.05% Tween-20) followed by Milli-Q ultrapure water and collected. For Western blotting analysis, beads were incubated with SDS-PAGE sample buffer (187.5 mM Tris–HCl [pH 6.8 at 25 °C], 6% w/v SDS, 30% glycerol, and 150 mM DTT) for 30 min to elute bound material. For MS analysis, magnetic beads were suspended in 100 mM NH_4_HCO_3_ solution, proteins were reduced with 5 mM DTT for 1 h, and alkylated with 15 mM iodoacetamide for 20 min in the dark. Protein digestion was carried with 0.1 μg sequencing-grade modified porcine trypsin (Promega) overnight at 37 °C. The reaction was stopped by the addition of trifluro acetic acid to a final concentration of 1% (v/v). Peptides were desalted with homemade stage tips (14) using Empore C18 disks (3M) before MS analysis.

### MS and Data Analysis

MS experiments were performed on an EASY nLC1000 nano-LC system connected to a QExactive mass spectrometer through a nanoelectrospray source EASYspray (Thermo Scientific). LC separations were carried out on an EASY column (C18, 2.0 μm beads, 100 Å, 75 μm inner diameter × 25 cm long, at 60 °C) (Thermo Scientific). The flow rate was 300 nl/min, and the solvent gradient was 2 to 30% solvent B for 120 min. Solvent A was aqueous 0.1% formic acid; and solvent B was 100% acetonitrile containing 0.1% formic acid. All solvents were MS-grade quality (Sigma). The mass spectrometer was operated in the data-dependent mode to automatically acquire sequence data in high-energy collision dissociation (HCD) cell for the ten most intense multiply charged. HCD fragmentation was set at a target value of 100,000 or maximum acquisition time of 100 ms. MS/MS scans were collected at 17,500 resolution in the Orbitrap cell. Additional MS conditions were electrospray voltage, 2.1 kV; no sheath or auxiliary gas flow, heated capillary temperature of 250 °C, and 25% normalized HCD collision energy. MS data were analyzed using MaxQuant (version 1.6.17.0) (https://www.maxquant.org/) (15) using UNIPROT human reviewed proteome database (downloaded in December 2020; 20,391 entries); carbamidomethyl (C+57) as a fixed modification, oxidation of methionine (M+16), acetylation (N-terminal+14) as variable modifications and up to two missed cleavages allowed. Precursor and fragment ion mass tolerance was 20 ppm. The false discovery rate (FDR) used was 1% at both the protein and the peptide level. Only proteins with two or more unique peptides were included in the final report. For NB4 or U937 cell lines, three replicates were run for each NTAL or isotype (negative control) IPs, and label-free quantification based on precursor ion intensities, according to intensity-based absolute quantification (iBAQ) approach (16), was performed. iBAQ intensities for proteins not detected in a particular run or experiments were adjusted to an arbitrary background intensity of 1000. The selection of NTAL interactors was based on the following criteria: (i) proteins identified with at least two unique peptides; (ii) detection of protein in four of the six NTAL IP experiments (3 NB4 + 3 U937); and (iii) iBAQ ratio (NTAL IP/control IP) in both NB4 and U937 experiments >30-fold. This stringent prioritization strategy intended to keep the list of potential interactors manageable, allowing the validation of several candidates, as well as to avoid secondary or transient interactions since NTAL is a scaffold protein.

### Protein–Protein Interactions and Functional Enrichment Analysis and Visualization

Protein–protein interactions and functional enrichment analysis and visualization were performed within Cytoscape (version 3.8.2) (http://www.cytoscape.org) (17). Interactor identifiers were mapped to human gene symbols and uploaded to the StringApp (Search Tool for the Retrieval of Interacting Genes/Protein) (version 1.6.0) to retrieve known protein–protein interactions (18). Gene Ontology (GO) analysis was performed using the Biological Networks Gene Ontology tool plugin (19) setting with overrepresentation, hypergeometric statistical test, Benjamini and Hochberg FDR correction, and significance level of 0.05.

Cytoscape plug-in Biological Networks Gene Ontology tool was used to perform the GO functional analysis involving the terms of cellular component for the identified selected interactors. An FDR *q* value <0.05 was used to be the threshold criterion for the selected GO conditions.

### Cell Fractionation

NB4 or U937 cells were washed twice with cold PBS and resuspended in buffer M (50 mM Hepes, pH 7.4, 10 mM NaCl, 5 mM MgCl_2_, 0.1 mM EDTA plus a protease inhibitor mixture, 1 mM Na_3_VO_4_, 1 mM NaF, and 1 mM Na_4_P_2_O_7_.10 dH_2_O) and broken by being passed through a 25-gauge needle 20 times and centrifuged at 500*g* for 10 min at 4 °C to pellet nuclei and intact cells. The supernatant was centrifuged at 16,000*g* for 20 min at 4 °C to pellet membranes. The pellets were resuspended in buffer A (25 mM MES (2-(*N*-morpholino)-ethanesulfonic acid), 150 mM NaCl, pH 6.5) and samples combined with an equal volume of buffer A containing 2% Triton X-100 and protease inhibitor. Samples were incubated on ice for 1 h and centrifuged at 16,000*g* for 20 min at 4 °C, and the supernatant (the Triton-soluble material) was designated as detergent-soluble membrane. Pellets were rinsed briefly with buffer A and resuspended in buffer B (10 mM Tris–Cl, pH 7.6, 150 mM NaCl, 60 mM β-octyl glucoside, and phosphatase and protease inhibitor). Samples were incubated on ice for 30 min and centrifuged at 16,000*g* for 20 min at 4 °C, and supernatants were collected as the lipid raft–enriched fraction that was designated as detergent-resistant membrane (DRM) (20).

### Confocal Microscopy

Cells were washed by centrifugation with PBS and placed on glass microscope slides coated with PBS containing Biobond (Electron Microscopy Sciences) at 37 °C for 30 min. Cells retained on the glass slides were fixed in 2% paraformaldehyde for 20 min at room temperature, washed and incubated with 0.1 M glycine for 15 min, and then blocked and permeabilized with 1% BSA containing 0.01% saponin for 30 min at room temperature. The slides were incubated with primary antibodies against target proteins for 2 h, then washed five times with PBS and incubated for 45 min with Alexa-Fluor 488 goat anti-mouse IgG antibody and Alexa Fluor-546 goat anti-rabbit IgG antibody (1:500) and then washed five times with PBS. Nuclei were stained with 4′,6-diamidino-2-phenylindole for 5 min. Slides were then mounted with a minimal volume of Prolong Gold Anti-Fade Reagent (#9071; Cell Signaling). Images were obtained by Laser scanning confocal imaging using a Leica SP8 confocal microscope (Leica Microsystems) operated by Leica Application Suite X (LAS X) software (Leica). Images were processed in an image-analysis program Fiji (12).

### Proximity Ligation Assay

The mouse/rabbit Duolink *In Situ* Orange Red Starter Kit (DUO92101; Sigma) was used for proximity ligation assay (PLA) assay. Cells were fixed, permeabilized, and probed with primary antibodies as described previously. Slides were washed and incubated (1 h, 37 °C) with specific plus and minus Duolink PLA probes (1:5). As a negative reaction control, slides were incubated only with Duolink PLA probes. After washing, slides were then incubated with ligation-ligase solution (30 min, 37 °C) followed by incubation with amplification-polymerase solution (2 h, 37 °C), according to the manufacturer's protocol. Slides were finally mounted with a minimal volume of Duolink Mounting medium containing 4′,6-diamidino-2-phenylindole (DUO82040; Sigma). Images were obtained by Laser scanning confocal imaging using a Leica SP5 confocal microscope (Leica) operated by Leica LAS AF Lite software (Leica). Images were processed in the Fiji image analysis program (12).

### Data Sources—PREdiction of Clinical Outcomes From Genomic Profiles Study

The PREdiction of Clinical Outcomes from Genomic Profiles (PRECOG) (21) was used to evaluate the association between NTAL and its interactors with OS in patients with AML. PRECOG comprehends eight different AML transcriptomic studies that included patients diagnosed with *de novo* AML, with age superior to 18 years old. All the patients included in the studies were treated with curative intent, according to Dutch–Belgian Hematology–Oncology Cooperative Group and the Swiss Group for Clinical Cancer Research. Statistical analyses were performed considering the expression levels for NTAL and its interactors as continuous variables.

### Gene Set Enrichment Analysis for NTAL Interactor Biological Pathways in AML

Gene set enrichment analysis (GSEA) was performed using the Broad Institute software (http://software.broadinstitute.org/gsea/index.jsp). All genes from the RNA-Seq of The Cancer Genome Atlas AML cohort were preranked according to their differential expression (fold change), and patients with AML (n = 121) were categorized into a high and low expression of NTAL interactors that were associated with differential OS, using their median expression rate as a cutoff. Enrichment scores were calculated based on Kolmogorov–Smirnov statistic, tested for significance using 1000 permutations, and normalized enrichment score to consider the size of each gene set. As suggested by GSEA, an FDR cutoff of 25% (FDR *q* value <0.25) was used (22).

### Statistical Analyses

All statistical analyses were performed using the SPSS (version 19.0, SPSS) and R (version 3.3.2, The Comprehensive R Archive Network project, www.r-project.org) software. All *p* values were two sided with a significance level of 0.05.

## Results

### NTAL KD Affects Akt Phosphorylation, Induces Apoptosis, and Decreases *in Vivo* Tumor Growth

**Fig. 1:**
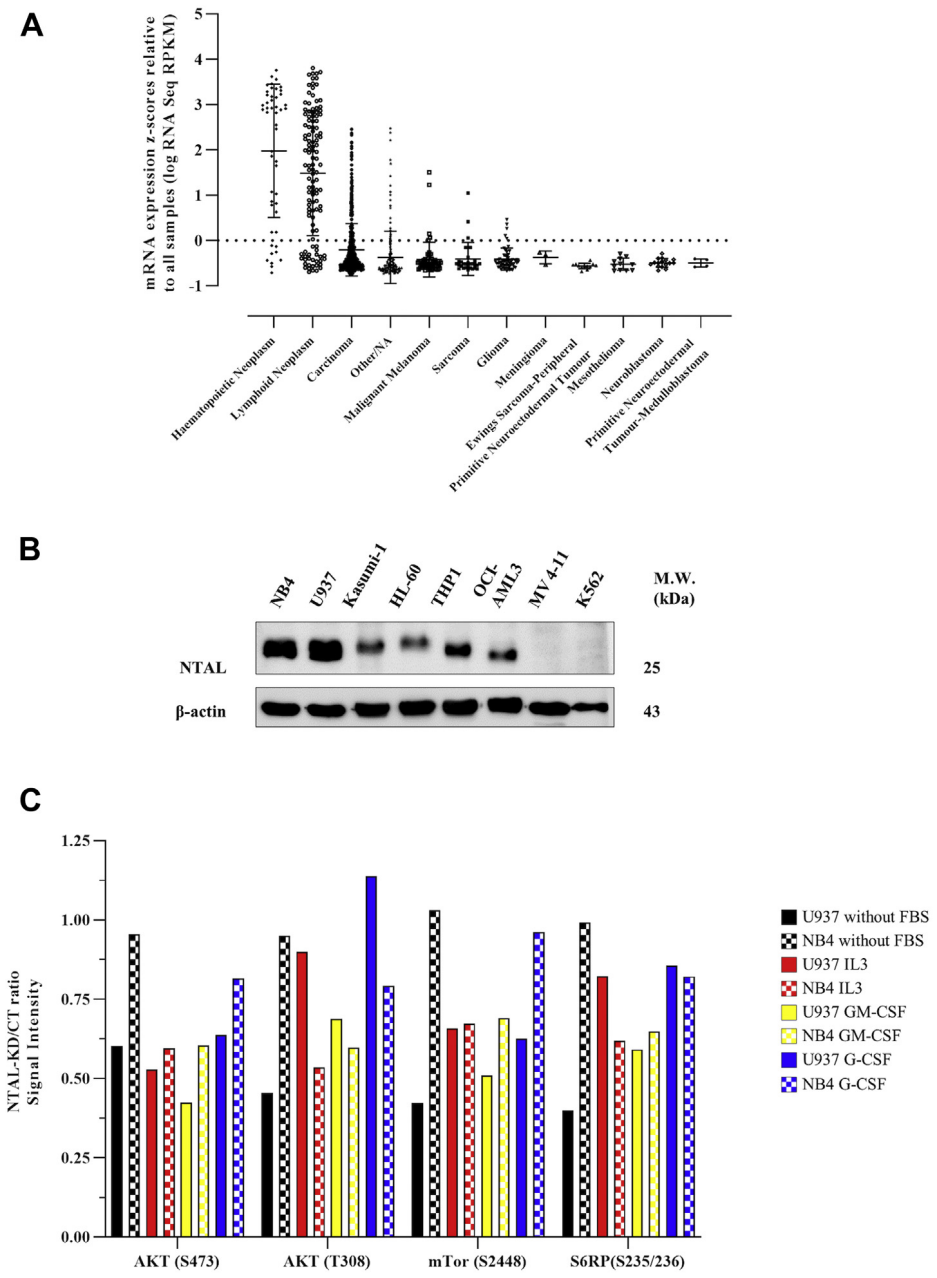
**NTAL expression in different leukemic strains and effect on downstream signaling.***A*, levels of NTAL RNA expression in several tissues of cancer by The Cancer Genome Atlas (TCGA). *B*, Western blotting analysis of NTAL protein extracts from various AML cell lines. *C*, effect of NTAL protein on the activation of the Akt pathway after the addition of MGF. NB4 (^∗^data published) (9) and U937 cells (CT or NTAL–KD) were cultured for 16 to 18 h in the absence of fetal bovine serum and MGF (hr-IL-3 or hr-GM-CSF or hr-G-CSF) was then added. Aliquots were withdrawn after 15 min of each treatment and evaluated with the PathScan Intracellular Signaling Array Kit. AML, acute myeloid leukemia; G-CSF granulocyte colony-stimulating factor; GM-CSF, granulocyte–macrophage colony-stimulating factor; hr, human recombinant; KD, knockdown; MGF, myeloid growth factor; NTAL, non–T cell activation linker.

NTAL, a lipid raft adaptor protein, was identified as an early mediator of alkylphospholipid antileukemic activity mediating Akt signaling (8). To further investigate the functional impact of NTAL in leukemia, we first examined The Cancer Cell Line Encyclopedia from the Broad Institute and Novartis (update 2019) to identify NTAL mRNA expression on several cancer cell lines. NTAL is particularly highly expressed in hematopoietic and neoplastic lymphoid cell lines (Fig. 1*A*). We also evaluated NTAL protein expression levels in eight different AML cell lines (Fig. 1*B*). Based on these results, we selected the two cell lines (NB4 and U937) with higher NTAL protein expression to further investigate NTAL relevance in AML models. The KD of NTAL in NB4 and U937 cells resulted in reduced levels of p-Akt (Ser-473 and Thr-308) proteins after serum deprivation and subsequent stimulation with myeloid growth factor, IL-3, granulocyte–macrophage CSF, or granulocyte CSF. A similar effect was observed for the downstream targets such as S6RP and mTOR (Fig. 1*C*). Thus, these results confirm previous findings and demonstrate that NTAL protein participates in the activation of the Akt pathway in both NB4 and U937 leukemia cell lines.

**Fig. 2:**
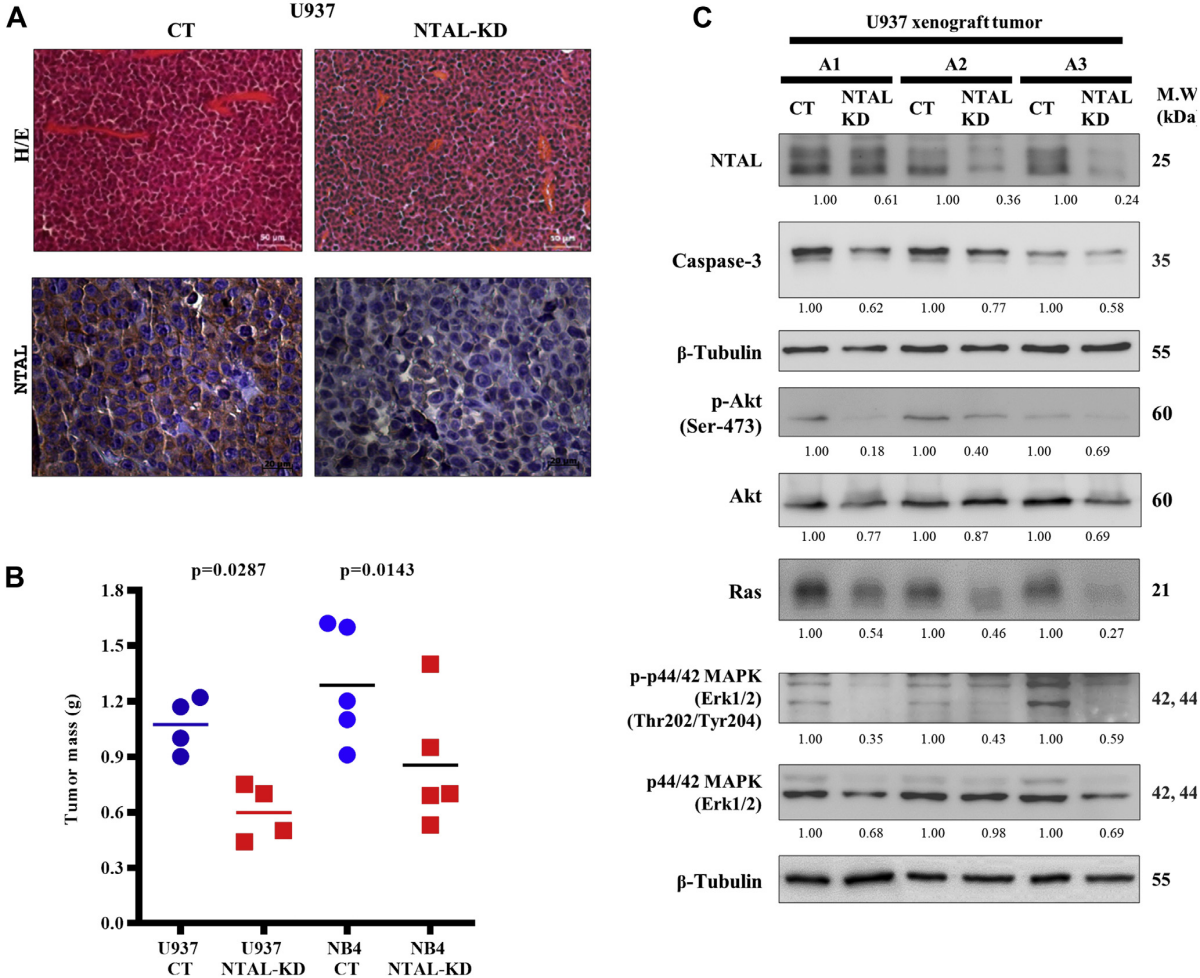
**Male 12-week-old NOD scid gamma mouse mice were injected subcutaneously into the left flank with 1 × 10**^**6**^**NTAL–KD cells and received an equal number of CT (shRNA negative control) cells in the right flank.***A*, sections of U937 (CT and NTAL–KD) tumors stained with hematoxylin and eosin or immunostained for NTAL. *B*, tumor masses derived after 2 weeks: U937: CT (1.073 g ± 0.1486) and NTAL–KD (0.5975 g ± 0.1506) (n = 4, *p* = 0.0287, 95% confidence interval) and NB4: CT (1.286 g ± 0.3137) and NTAL–KD (0.8540 g ± 0.3402) (n = 5, *p* = 0.0143, 95% confidence interval) (^∗^data published) (9). *C*, Western blotting analysis of total tumor protein extracts from engrafted tumors for NTAL, procaspase-3, p-Akt (Ser-473), Akt, Ras, p-p44/42 MAPK, and p44/42 MAPK (animals A1 = 1, A2 = 2, and A3 = 3). 30 μg of tumor total extract were loaded in each gel lane. CT, control; KD, knockdown; MAPK, mitogen-activated protein kinase; NTAL, non–T cell activation linker.

We also sought to demonstrate the participation of NTAL in cell proliferation and in the activation of apoptosis *in vivo.* For that, we evaluated how NTAL–KD affects leukemic cell lines NB4 and U937 xenograft tumor growth in NOD scid gamma mouse mice. No histological differences were observed between xenograft tumors formed by control and NTAL–KD (Fig. 2*A*). However, NTAL–KD tumors presented a lower growth rate, and the mean tumor weight was reduced by 1.8-fold for U973 NTAL–KD and 1.5-fold for NTAL–KD NB4 (Fig. 2*B*) (9). Also, the evaluation of proliferation and apoptotic markers in NTAL–KD xenograft tumors showed decreased levels of procaspase-3, p-Akt (Ser-473), Akt, Ras, p-p44/42 mitogen-activated protein kinase (MAPK), and total p44/42 MAPK proteins. We also confirmed the reduced levels of NTAL protein in tumor samples by Western blotting (Fig. 2*C*).

### Identification of New NTAL Interactors in AML Cells

**Fig. 3:**
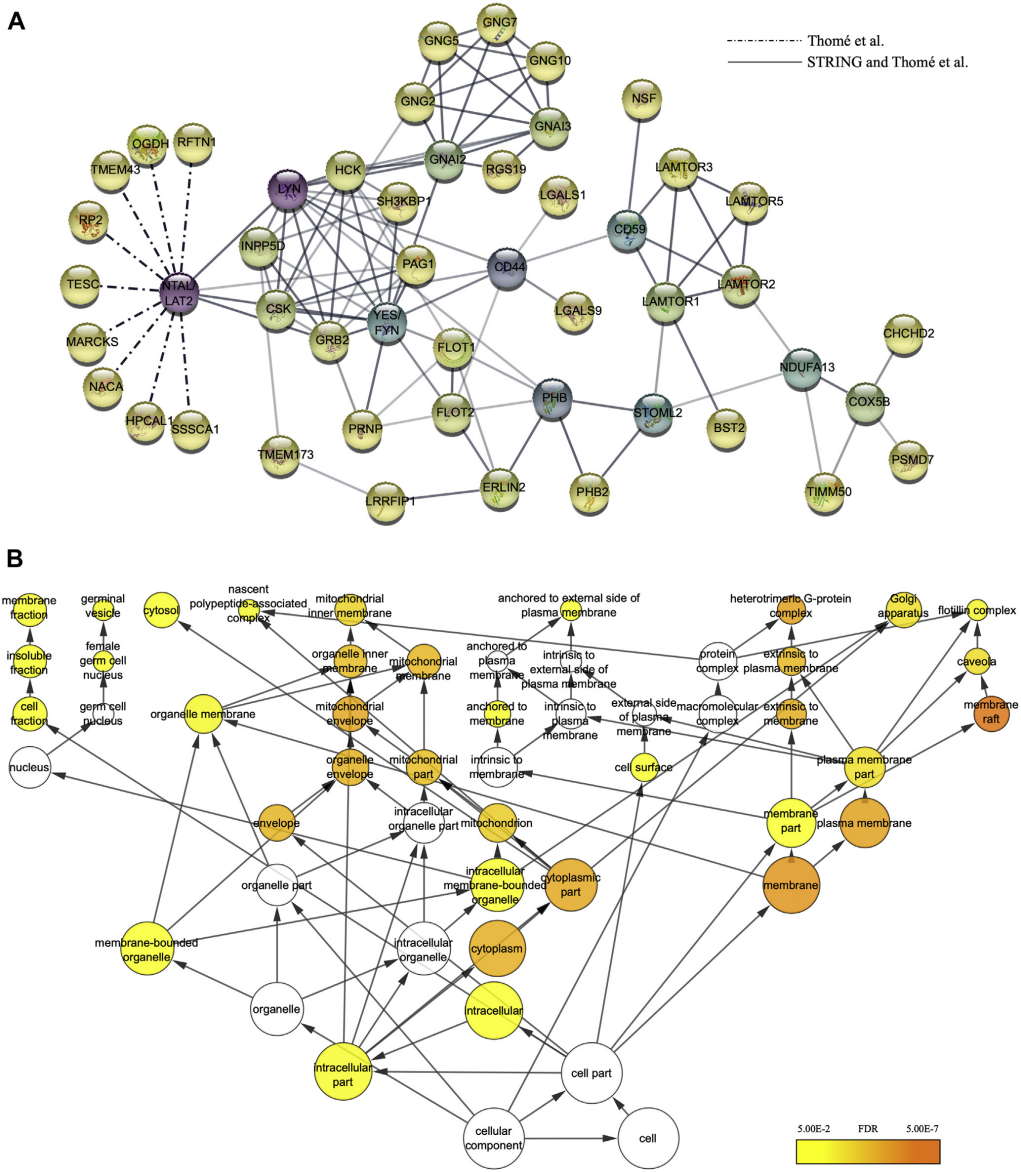
**Bioinformatics analysis of NTAL protein interactors identified by IP–MS/MS (IP/MS).***A*, STRING (https://string-db.org/) and Cytoscape software were used for the functional analysis of protein–protein interactions. Proteins in *purple*, *blue*, and *green* spheres represent the first shelf of protein interactions, whereas proteins in *yellow* spheres represent the second shelf of interactors. *Dashed lines* indicates protein interactions only described by the current study, whereas *continuous lines* represent protein interactions that were previously described and also observed in the current study. *B*, GO analysis of NTAL interactors identified by MS analysis in NB4 and U937 cells was performed using BiNGO analysis of Cytoscape software. *Yellow circles* represent the GO terms statistically significant. GO terms for cellular component (CC) with a statistical significance of FDR *q* value <0.05 were considered for further conclusions. BiNGO, Biological Networks Gene Ontology; FDR, false discovery rate; GO, Gene Ontology; IP, immunoprecipitation; NTAL, non–T cell activation linker.

To provide more details on the impact of NTAL in AML cell proliferation and apoptosis, we aimed to identify a new proteome-wide network of NTAL interactors. The evaluation of NTAL protein expression in different AML cell lines (Fig. 1*B*) leads to the selection of NB4 and U937 as NTAL highly expressing cells line for further experiments. IP of NTAL and bound interactors followed by identification by LC–MS/MS and label-free quantification (iBAQ) revealed a long list of proteins with less than 1% FDR (supplemental Table S2). This extensive list of possible interactors was prioritized based on the relative abundance of proteins observed with NTAL antibody IP compared with the isotype control IP, eliminating potential nonspecific proteins as well as secondary or transient interactions, since NTAL is a scaffold protein. Forty-nine proteins were selected as potential interactors with NTAL based on the detection in both leukemic cell lines (Table 1; Fig. 3*A*). Among the described interactors, Grb2, SHIP1/INPP5D, Lyn, Fyn/Yes, and Hck have been independently identified as potential interactors of NTAL (2, 23, 24, 25, 26), which supported our results.

**Table 1:** Identification of NTAL protein interactors by IP–MS/MS

Entry	Gene names	Protein	NTAL interactors' rank	NTAL/CT		Subcellular location (CC)
				NB4	U937	
P13987	CD59, MIC11, MIN1, MIN2, MIN3, MSK21	CD59 glycoprotein (1F5 antigen) (20 kDa homologous restriction factor) (HRF-20) (HRF20) (MAC-inhibitory protein) (MAC-IP) (MEM43 antigen) (Membrane attack complex inhibition factor) (MACIF) (Membrane inhibitor of reactive lysis) (MIRL) (Protectin) (CD antigen CD59)	1	11003.31	65.79	Cell membrane; lipid anchor. GPI anchor Secreted
P63218	GNG5, GNGT5	Guanine nucleotide-binding protein G(I)/G(S)/G(O) subunit gamma-5	2	8234.27	3375.9	Cell membrane; lipid anchor; cytoplasmic side
O43504	LAMTOR5, HBXIP, XIP	Ragulator complex protein LAMTOR5 (Hepatitis B virus X-interacting protein) (HBV X-interacting protein) (HBX-interacting protein) (Late endosomal/lysosomal adaptor and MAPK and MTOR activator 5)	3	7832.41	417.52	Lysosome Cytoplasm, cytosol
Q6IAA8	LAMTOR1, C11orf59, PDRO, PP7157	Ragulator complex protein LAMTOR1 (Late endosomal/lysosomal adaptor and MAPK and MTOR activator 1) (Lipid raft adaptor protein p18) (Protein associated with DRMs and endosomes) (p27Kip1-releasing factor from RhoA) (p27RF-Rho)	4	7359.82	195.24	Late endosome membrane; lipid anchor; cytoplasmic side Lysosome membrane; lipid anchor; cytoplasmic side Cell membrane
Q9Y6H1	CHCHD2, C7orf17, AAG10	Coiled-coil-helix-coiled-coil-helix domain-containing protein 2 (Aging-associated gene 10 protein) (HCV NS2 trans-regulated protein) (NS2TP)	5	5420.04	584.03	Nucleus Mitochondrion Mitochondrion intermembrane space
P49795	RGS19, GAIP, GNAI3IP	Regulator of G-protein signaling 19 (RGS19) (G-alpha-interacting protein) (GAIP)	6	5149.09	1959.82	Membrane; lipid anchor
O60262	GNG7, GNGT7	Guanine nucleotide-binding protein G(I)/G(S)/G(O) subunit gamma-7	7	4588.96	3036.13	Cell membrane; lipid anchor; cytoplasmic side
Q9Y2Q5	LAMTOR2, MAPBPIP, ROBLD3, HSPC003	Ragulator complex protein LAMTOR2 (Endosomal adaptor protein p14) (Late endosomal/lysosomal Mp1-interacting protein) (Late endosomal/lysosomal adaptor and MAPK and MTOR activator 2) (Mitogen-activated protein-binding protein-interacting protein) (MAPBP-interacting protein) (Roadblock domain-containing protein 3)	8	4507.38	59.02	Late endosome membrane; peripheral membrane protein; cytoplasmic side Lysosome membrane; peripheral membrane protein; cytoplasmic side.
Q9UHA4	LAMTOR3, MAP2K1IP1, MAPKSP1, PRO2783	Ragulator complex protein LAMTOR3 (Late endosomal/lysosomal adaptor and MAPK and MTOR activator 3) (MEK-binding partner 1) (Mp1) (Mitogen-activated protein kinase kinase 1-interacting protein 1) (Mitogen-activated protein kinase scaffold protein 1)	9	3491.67	170.53	Late endosome membrane; peripheral membrane protein; cytoplasmic side
Q14699	RFTN1, KIAA0084, MIG2	Raftlin (Cell migration-inducing gene 2 protein) (Raft-linking protein)	10	2865.07	1337.46	Cell membrane; lipid anchor Cytoplasm Membrane raft Endosome Early endosome
O60232	ZNRD2, SSSCA1	Protein ZNRD2 (Autoantigen p27) (Sjoegren syndrome/scleroderma autoantigen 1) (Zinc ribbon domain-containing protein 2)	11	2363.64	2000.01	
Q10589	BST2	Bone marrow stromal antigen 2 (BST-2) (HM1.24 antigen) (Tetherin) (CD antigen CD317)	12	2207.63	465.4	Golgi apparatus, trans-Golgi network Cell membrane; single-pass type II membrane protein Cell membrane; lipid anchor, GPI anchor Membrane raft Cytoplasm Apical cell membrane; Golgi apparatus, trans-Golgi network Late endosome
Q9NWQ8	PAG, CBP, PAG	Phosphoprotein associated with glycosphingolipid-enriched microdomains 1 (Csk-binding protein) (Transmembrane adapter protein PAG) (Transmembrane phosphoprotein Cbp)	13	1932.26	1251.04	Cell membrane; single-pass type III membrane protein
P10606	COX5B	Cytochrome c oxidase subunit 5B. mitochondrial (Cytochrome c oxidase polypeptide Vb)	14	1396.1	71.9	Mitochondrion inner membrane; peripheral membrane protein; matrix side
P41240	CSK	Tyrosine-protein kinase CSK (EC 2.7.10.2) (C-Src kinase) (Protein-tyrosine kinase CYL)	15	1391.21	947.27	Cytoplasm Cell membrane
P29966	MARCKS, MACS, PRKCSL	Myristoylated alanine-rich C-kinase substrate (MARCKS) (Protein kinase C substrate. 80 kDa protein. light chain) (80K-L protein) (PKCSL)	16	1304.47	643.21	Cytoplasm, cytoskeleton Membrane, lipid anchor
P16070	CD44, LHR, MDU2, MDU3, MIC4	CD44 antigen (CDw44) (Epican) (Extracellular matrix receptor III) (ECMR-III) (GP90 lymphocyte homing/adhesion receptor) (HUTCH-I) (Heparan sulfate proteoglycan) (Hermes antigen) (Hyaluronate receptor) (Phagocytic glycoprotein 1) (PGP-1) (Phagocytic glycoprotein I) (PGP-I) (CD antigen CD44)	17	768.65	1810.18	Cell membrane; single-pass type I membrane protein Cell projection, microvillus
O75695	RP2	Protein XRP2	18	620.41	280.05	Cell membrane; lipid anchor; cytoplasmic side Cell projection, cilium {ECO: 0000269|PubMed: 20106869}
P04156	PRNP, ALTPRP, PRIP, PRP	Major prion protein (PrP) (ASCR) (PrP27–30) (PrP33–35C) (CD antigen CD230)	19	605.57	213.25	Cell membrane; lipid anchor, GPI anchor, Golgi apparatus
Q9GZY6	LAT2, LAB, NTAL, WBS15, WBSCR15, WBSCR5, HSPC046	Linker for activation of T-cells family member 2 (Linker for activation of B-cells) (Membrane-associated adapter molecule) (Non-T-cell activation linker) (Williams-Beuren syndrome chromosomal region 15 protein) (Williams-Beuren syndrome chromosomal region 5 protein)	20	601.91	23203.65	Cell membrane; single-pass type III membrane protein
Q9P0J0	NDUFA13, GRIM19, CDA016, CGI-39	NADH dehydrogenase [ubiquinone] 1 alpha subcomplex subunit 13 (Cell death regulatory protein GRIM-19) (Complex I-B16.6) (CI-B16.6) (Gene associated with retinoic and interferon-induced mortality 19 protein) (GRIM-19) (Gene associated with retinoic and IFN-induced mortality 19 protein) (NADH-ubiquinone oxidoreductase B16.6 subunit)	21	560.22	31.04	Mitochondrion inner membrane; single-pass membrane protein; matrix side Nucleus
Q9BTV4	TMEM43, UNQ2564/PRO6244	Transmembrane protein 43 (Protein LUMA)	22	558.34	30.56	Endoplasmic reticulum Nucleus inner membrane; Multipass membrane protein
Q96BS2	TESC, CHP3	Calcineurin B homologous protein 3 (Tescalcin) (TSC)	23	547.81	130.58	Nucleus Cytoplasm Membrane; lipid anchor Cell membrane Cell projection, lamellipodium Cell projection, ruffle membrane
P37235	HPCAL1, BDR1	Hippocalcin-like protein 1 (Calcium-binding protein BDR-1) (HLP2) (Visinin-like protein 3) (VILIP-3)	24	547.8	283.07	Membrane; lipid anchor
O75955	FLOT1	Flotillin-1	25	401.21	381.53	Cell membrane; peripheral membrane protein Endosome Membrane, caveola; peripheral membrane protein Melanosome Membrane raft Membrane-associated protein of caveola
P59768	GNG2	Guanine nucleotide-binding protein G(I)/G(S)/G(O) subunit gamma-2 (G gamma-I)	26	196.72	4926.98	Cell membrane; lipid anchor; cytoplasmic side
Q02218	OGDH	2-oxoglutarate dehydrogenase. mitochondrial (EC 1.2.4.2) (2-oxoglutarate dehydrogenase complex component E1) (OGDC-E1) (Alpha-ketoglutarate dehydrogenase)	27	131.05	87.76	Mitochondrion matrix Nucleus
Q96B97	SH3KBP1 CIN85	SH3 domain-containing kinase-binding protein 1 (CD2-binding protein 3) (CD2BP3) (Cbl-interacting protein of 85 kDa) (Human Src family kinase-binding protein 1) (HSB-1)	28	126.09	147.21	Cytoplasm, cytoskeleton Cytoplasmic vesicle membrane; peripheral membrane protein Cell junction, synapse, synaptosome Cell junction, focal adhesion
P51665	PSMD7, MOV34L	26S proteasome non-ATPase regulatory subunit 7 (26S proteasome regulatory subunit RPN8) (26S proteasome regulatory subunit S12) (Mov34 protein homolog) (Proteasome subunit p40)	29	124.02	484.03	
E9PAV3	NACA	Nascent polypeptide-associated complex subunit alpha. muscle-specific form (Alpha-NAC. muscle-specific form) (skNAC)	30	107.67	37.21	Cytoplasm Nucleus
Q32MZ4	LRRFIP1, GCF2, TRIP	Leucine-rich repeat flightless-interacting protein 1 (LRR FLII-interacting protein 1) (GC-binding factor 2) (TAR RNA-interacting protein)	31	90.86	212.84	Nucleus Cytoplasm
P07948	LYN, JTK8	Tyrosine-protein kinase Lyn (EC 2.7.10.2) (Lck/Yes-related novel protein tyrosine kinase) (V-yes-1 Yamaguchi sarcoma viral related oncogene homolog) (p53Lyn) (p56Lyn)	32	75.97	2664.86	Cell membrane Nucleus, cytoplasm Cytoplasm, perinuclear region Golgi apparatus Membrane; lipid anchor
P09382	LGALS1	Galectin-1 (Gal-1) (14 kDa laminin-binding protein) (HLBP14) (14 kDa lectin) (Beta-galactoside-binding lectin L-14-I) (Galaptin) (HBL) (HPL) (Lactose-binding lectin 1) (Lectin galactoside-binding soluble 1) (Putative MAPK-activating protein PM12) (S-Lac lectin 1)	33	75.23	68.21	Secreted, extracellular space, extracellular matrix Cytoplasm Secreted
O00182	LGALS9	Galectin-9 (Gal-9) (Ecalectin) (Tumor antigen HOM-HD-21)	34	74.99	19304.74	Cytoplasm Nucleus Secreted
P35232	PHB, PHB1	Prohibitin	35	73.81	1119.05	Mitochondrion inner membrane Nucleus Cytoplasm Cell membrane
P62993	GRB2, ASH	Growth factor receptor-bound protein 2 (Adapter protein GRB2) (Protein Ash) (SH2/SH3 adapter GRB2)	36	50.54	170.66	Nucleus Endosome Golgi apparatus
Q3ZCQ8	TIMM50, TIM50, PRO1512	Mitochondrial import inner membrane translocase subunit TIM50	37	47.56	164.48	Mitochondrion inner membrane; single-pass membrane protein
P08631	HCK	Tyrosine-protein kinase HCK (EC 2.7.10.2) (Hematopoietic cell kinase) (Hemopoietic cell kinase) (p59-HCK/p60-HCK) (p59Hck) (p61Hck)	38	43.81	1414.6	Lysosome Membrane; lipid anchor, podosome membrane; lipid anchor Cytosol Cell membrane; lipid anchor Caveola; lipid anchor Focal adhesion Cytoskeleton Golgi apparatus Cytoplasmic vesicle Nucleus
0P08754	GNAI3	Guanine nucleotide-binding protein G(i) subunit alpha-3 (G(i) alpha-3)	39	41.1	14540.08	Cytoplasm Cell membrane; lipid anchor Cytoplasm, cytoskeleton, microtubule-organizing center, centrosome
Q99623	PHB2, BAP, REA	Prohibitin-2 (B-cell receptor-associated protein BAP37) (D-prohibitin) (Repressor of estrogen receptor activity)	40	40.77	1174.56	Mitochondrion inner membrane Cytoplasm Nucleus Cell membrane
P04899	GNAI2, GNAI2B	Guanine nucleotide-binding protein G(i) subunit alpha-2 (Adenylate cyclase-inhibiting G alpha protein)	41	37.68	479.24	Cytoplasm Cytoplasm, cytoskeleton, microtubule-organizing center, centrosome Cell membrane Membrane; lipid anchor
Q9UJZ1	STOML2,SLP2, HSPC108	Stomatin-like protein 2. mitochondrial (SLP-2) (EPB72-like protein 2) (Paraprotein target 7) (Paratarg-7)	42	37.29	37.06	Cell membrane; peripheral membrane Mitochondrion Mitochondrion inner membrane; lipid anchor Mitochondrion intermembrane space Membrane raft Cytoplasm, cytoskeleton
P06241	FYN	Tyrosine-protein kinase Fyn (EC 2.7.10.2) (Proto-oncogene Syn) (Proto-oncogene c-Fyn) (Src-like kinase) (SLK) (p59-Fyn)	43	36.63	764.61	Cytoplasm, nucleus, cell membrane
P50151	GNG10, GNGT10	Guanine nucleotide-binding protein G(I)/G(S)/G(O) subunit gamma-10	44	35.11	2135.77	Cell membrane; lipid anchor; cytoplasmic side
Q92835	INPP5D SHIP SHIP1	Phosphatidylinositol 3.4.5-trisphosphate 5-phosphatase 1 (EC 3.1.3.86) (Inositol polyphosphate-5-phosphatase D) (EC 3.1.3.56) (Inositol polyphosphate-5-phosphatase of 145 kDa) (SIP-145) (Phosphatidylinositol 4.5-bisphosphate 5-phosphatase) (EC 3.1.3.36) (SH2 domain-containing inositol 5′-phosphatase 1) (SH2 domain-containing inositol phosphatase 1) (SHIP-1) (p150Ship) (hp51CN)	45	33.69	1315.36	Cytoplasm {ECO: 0000269|PubMed: 10822173} Cell membrane; peripheral membrane protein Membrane raft Cytoplasm. cytoskeleton Membrane; peripheral membrane protein
Q86WV6	STING1, ERIS, MITA, TMEM173	Stimulator of interferon genes protein (hSTING) (Endoplasmic reticulum interferon stimulator) (ERIS) (Mediator of IRF3 activation) (hMITA) (Transmembrane protein 173)	46	32.51	788.15	Endoplasmic reticulum membrane; multipass membrane protein Cytoplasm. perinuclear region Endoplasmic reticulum–Golgi intermediate compartment membrane; multipass membrane protein Cytoplasmic vesicle. autophagosome membrane; multipass membrane protein Mitochondrion outer membrane; multipass membrane protein Cell membrane; multipass membrane protein
P46459	NSF	Vesicle-fusing ATPase (EC 3.6.4.6) (N-ethylmaleimide-sensitive fusion protein) (NEM-sensitive fusion protein) (Vesicular-fusion protein NSF)	47	32.17	103.61	Cytoplasm
O94905	ERLIN2, C8orf2, SPFH2, UNQ2441/PRO5003/PRO9924	Erlin-2 (Endoplasmic reticulum lipid raft-associated protein 2) (Stomatin-prohibitin-flotillin-HflC/K domain-containing protein 2) (SPFH domain-containing protein 2)	48	30.54	1786.4	Endoplasmic reticulum membrane; single-pass type II membrane protein
Q14254	FLOT2, ESA1, M17S1	Flotillin-2 (Epidermal surface antigen) (ESA) (Membrane component chromosome 17 surface marker 1)	49	30.45	1008.94	Cell membrane; peripheral membrane protein Membrane, caveola; peripheral membrane protein, endosome Membrane; lipid anchor

Forty-nine proteins were selected as potential interactors with NTAL in leukemic cell lines (NB4 and U937). GO term obtained from UNIPROT knowledgebase annotation.

### Analysis of the Subcellular Localization of NTAL Interactors

**Fig. 4:**
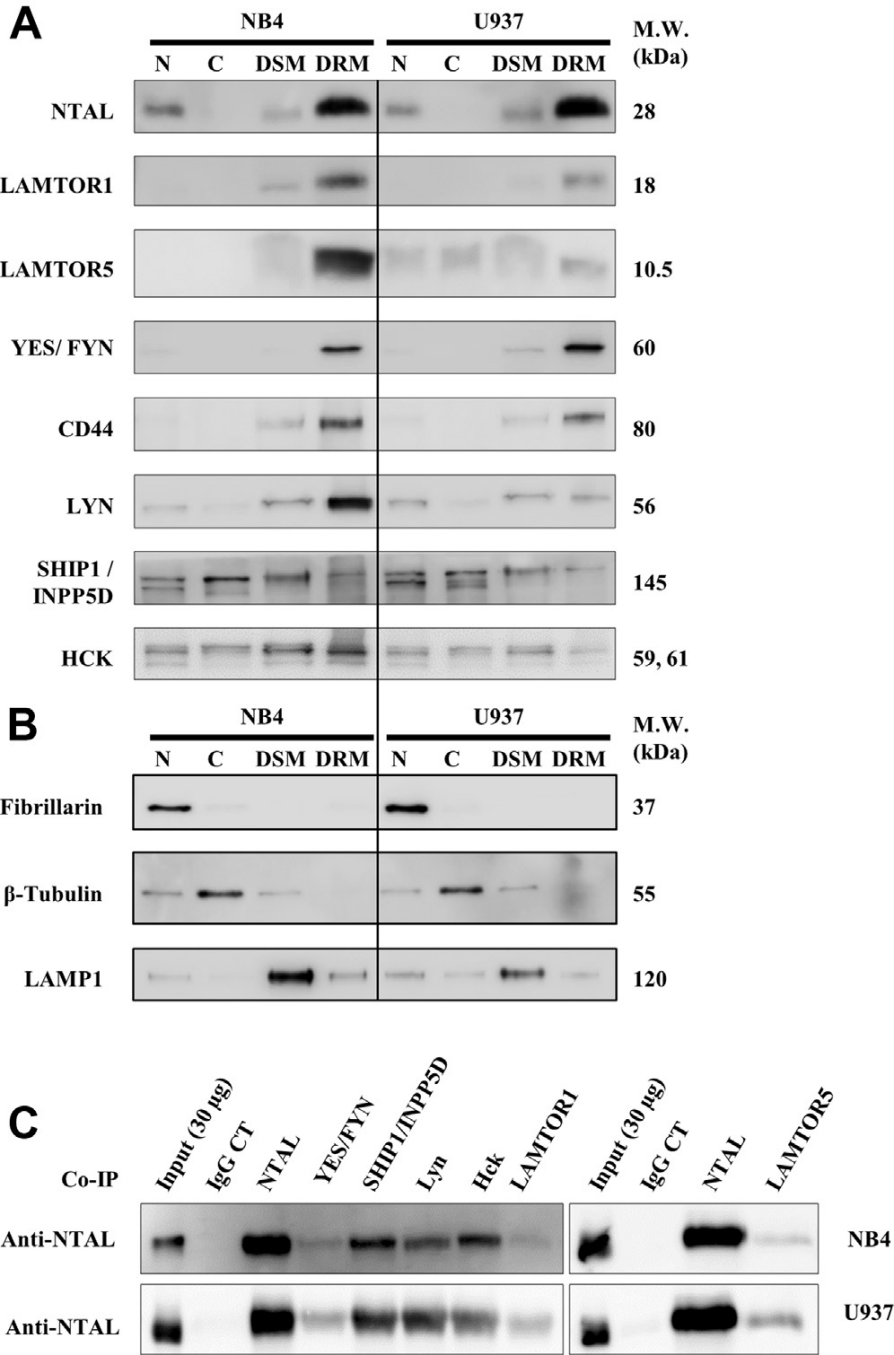
**Localization of NTAL interactors.***A*, isolation of nuclei (N), cytosol (C), detergent-resistant membrane (DRM), and detergent-soluble membrane (DSM) fractions from NB4 and U937 cells, prepared by differential detergent solubility followed by Western blotting (NTAL, LAMTOR1, LAMTOR5, Fyn/Yes, CD44, Lyn, SHIP1/INPP5D, and Hck). *B*, quality control Western blotting of specific markers for nonraft membranes, cytosol, and nuclear proteins. β-tubulin, appears in the cytosol fraction; LAMP1, lysosome-associated membrane protein 1, appears in the nonraft membranes; and fibrillarin is present only in the nuclear fraction. *C*, NTAL interactors were validated by counter-IP (co-IP) using antibodies against some of the identified proteins, followed by immunolabeling using NTAL antibody. IgG represents isotype negative controls for each experiment. IP, immunoprecipitation; NTAL, non–T cell activation linker.

From the list of 49 NTAL interactors, we found that most proteins were associated with the “cell membrane” and “lipid raft” cellular component terms (Fig. 3*B*; Table 1). To further demonstrate the cellular localization of NTAL interactors, we examined several proteins in NB4 and U937 cellular fractions enriched for nuclei (N), cytosol (C), detergent-soluble membrane, or DRM obtained by differential detergent solubility (20). Western blotting confirmed that LAMTOR1, LAMTOR5, Fyn/Yes, CD44, Lyn, SHIP1/INPP5D, and Hck in the DRM lipid raft–rich fraction together with NTAL (Fig. 4, *A* and *B*). SHIP1/INPP5D and the kinase Hck were detected in all four subcellular fractions, suggesting their presence in multiple cellular compartments.

**Fig. 5:**
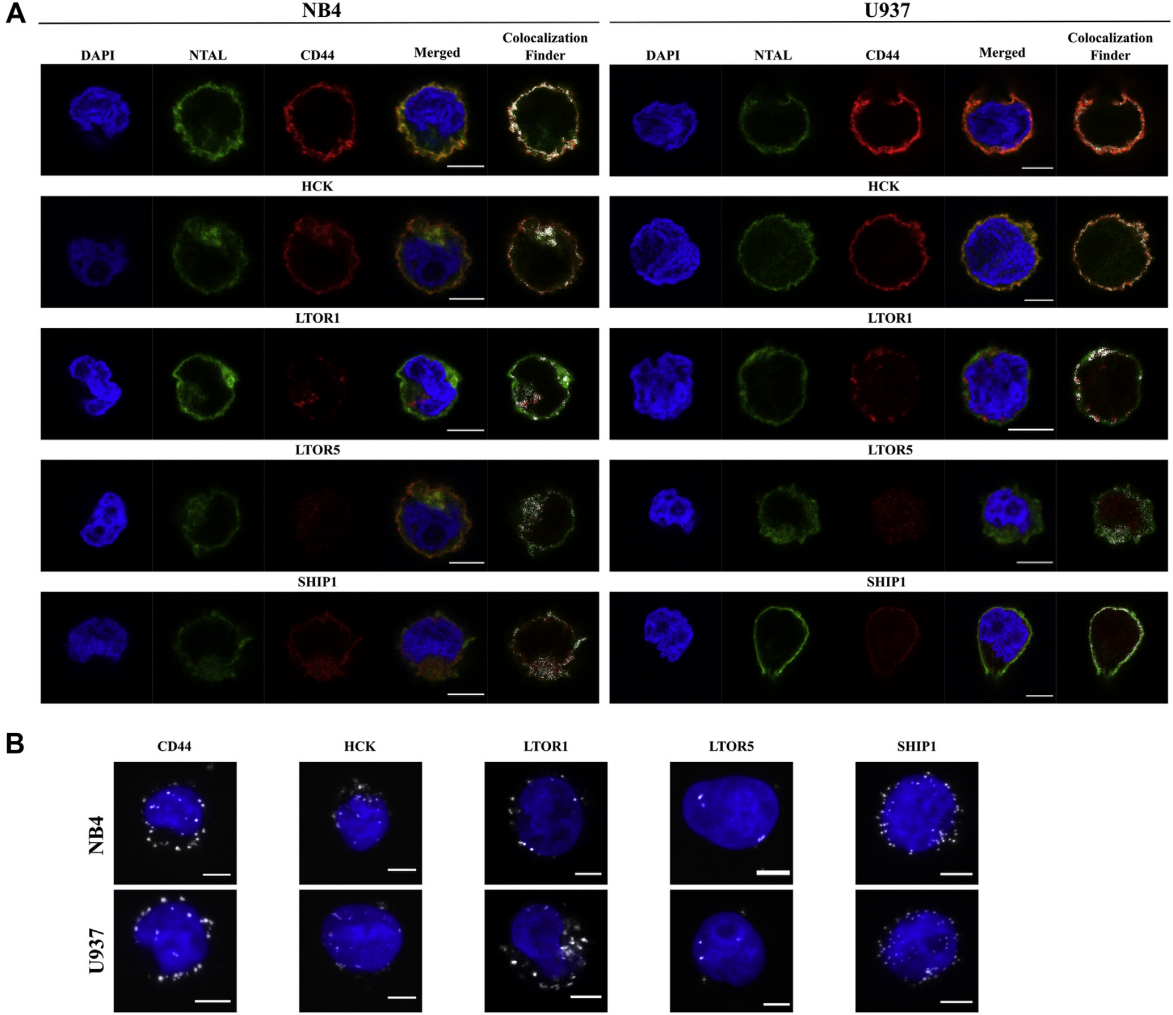
**Validation of the collocation of NTAL and its interactors.***A*, colocalization of proteins NTAL and CD44 or Hck or LAMTOR1 or LAMTOR5 or SHIP1/INPP5D. Labeling was performed with anti-NTAL antibodies (*green*) and anti-CD44 (*red*) or anti-Hck (*red*) or anti-LAMTOR1 (*red*) or anti-LAMTOR5 (*red*) or SHIP1/INPP5D (*red*) antibodies. The merged images show regions of colocalization based on the ImageJ plugin Colocalization Finder. *B*, proximity ligation assays (PLAs) show interactions between NTAL and CD44 or Hck or LAMTOR1 or LAMTOR5 or SHIP1/INPP5D as *white spots*, equivalent to one molecular interaction. Images were acquired by confocal microscopy. Confocal microscopy and PLA were performed in the NB4 and U937 cells. NTAL, non–T cell activation linker.

Several proteins (Fyn/Yes, SHIP1/INPP5D, Lyn, Hck, LAMTOR1, and LAMTOR5) had their interactions with NTAL confirmed by counter-IPs (Fig. 4*C*). Colocalization of NTAL with CD44, Hck, LAMTOR1, LAMTOR5, or SHIP1/INPP5D was further demonstrated by confocal microscopy and PLA (Fig. 5). As shown in Figure 5*A*, staining for NTAL was observed in the plasma membrane of both cell lines and colocalized with its interactors close to the membrane. The formation of the NTAL–CD44 or NTAL–Hck or NTAL–LAMTOR1 or NTAL–LAMTOR5 or NTAL–SHIP1/INPP5D complexes close to the plasma membrane was also demonstrated by PLA, which shows interactions within 40 nm of distance (Fig. 5*B*). Altogether, the data support the participation of NTAL as an adaptor or scaffold protein that retains several interactions close to the intracellular side of the cellular membrane into lipid raft structures.

### The Molecular Signature of NTAL and Its Interactors Is Associated With L-GMP Cells

**Fig. 6:**
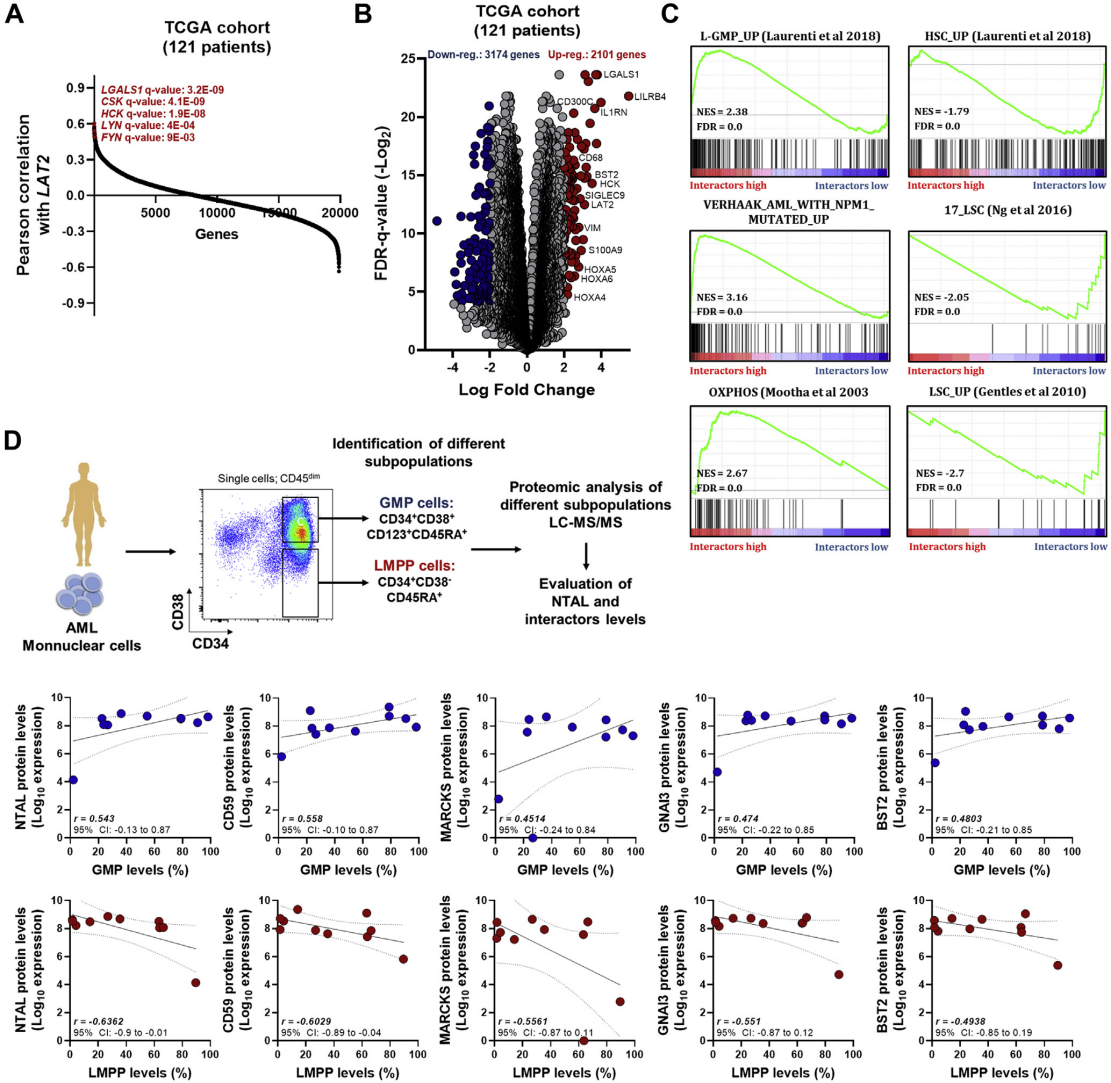
**Differential expression of NTAL interactors defines different biology in patients with AML.***A*, Pearson correlation shows the positively correlated genes with LAT2 (NTAL) gene in patients with AML (TCGA cohort). *B*, volcano plot of differentially expressed genes (unsupervised clustering) in AML samples from TCGA cohort dichotomized according to the gene expression of NTAL interactors (dichotomization point: median value; groups: high and low expression of NTAL interactors associated with overall survival). *C*, GSEA on a preranked gene list based on the leading-edge genes for NTAL interactor expression in 121 *de novo* AML patient samples from TCGA study. Genes were ranked based on Pearson's correlation with NTAL interactor gene expression. Normalized enrichment score (NES) and false discovery rate (FDR) were used for significance. *D*, Pearson correlation between the protein expression levels of NTAL and its interactors and the levels of the different subcompartments (GMPs and LMPP, defined as described in the *upper chart*) in patients with AML. AML, acute myeloid leukemia; GMP, granulocyte–macrophage progenitor; GSEA, gene set enrichment analysis; LAT2, linker for activation of T cells 2; LMPP, lymphoid-primed multipotent progenitor; NTAL, non–T cell activation linker; TCGA, The Cancer Genome Atlas.

**Fig. 7:**
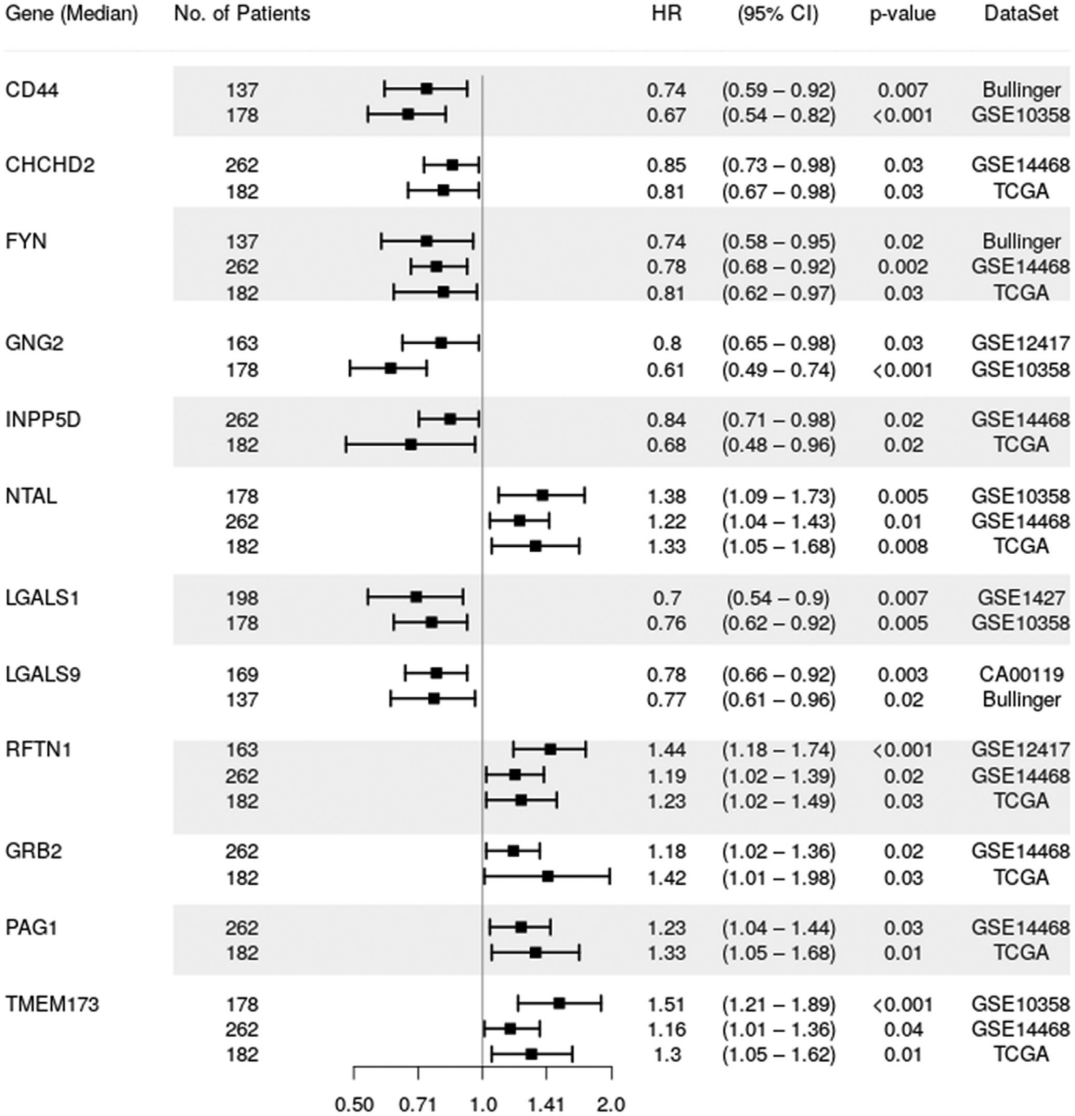
**Forest plot for multivariable analysis identified 12 NTAL protein interactors as independent predictors of overall survival in patients with AML.** Cox hazard proportional model for overall survival of NTAL protein interactor gene expression signatures retrieved from PRECOG database (21). Hazard ratio (HR) >1 indicates that increasing values for continuous variable or the first factor for categorical variables has the poorer outcome. HR and their respective 95% confidence interval (95% CI) are indicated with *black squares* and a *line*, respectively. Public datasets used for comparisons were described by the title of gene series expression (GSE) or by the code of the study previously published (28). AML, acute myeloid leukemia; NTAL, non–T cell activation linker; PRECOG, PREdiction of Clinical Outcomes from Genomic Profiles.

Next, we analyzed the expression of the molecular signature associated with NTAL and its 49 interactors (NTAL network) (Table 1) in AML samples from The Cancer Genome Atlas cohort (27). Pearson correlation showed that several genes that encoded in the NTAL protein network exhibited a high positive correlation with NTAL gene (LAT2) in patients with AML (Fig. 6*A*). Clustered transcriptome analysis demonstrated distinguished gene expression signature between patients with high *versus* low NTAL network expression (Fig. 6*B*). Interestingly, patients with high NTAL interactor expression were associated with increased expression of HOXA, HOXB, RAS, and AKT1 genes (Fig. 6*B*). Subsequently, GSEA associated NTAL interactors of high AML patients with the terms “Oxidative phosphorylation metabolism,” “L-GMP signature” and “Up-regulated genes in AML patients with NPM1 mutant,” whereas interactors of the low AML patients were associated with “HSC-like signature” and “17 Leukemic stem cell genes” (Fig. 6*C*). Using the previously published proteomic dataset of a well characterized AML cohort (28), we noticed that AML patients with an immunophenotypic profile characteristic for GMP-Like AML presented increased NTAL and its interactor protein levels when compared with AML samples with a more /HSC-like immunophenotype (Fig. 6*D*). Taken together, these results suggest that AML patients with high levels of NTAL and its interactors differ significantly in their biology compared with patients with low NTAL network expression. Finally, using the public databank BloodSpot (29), the expression of NTAL and its interactors was evaluated in healthy BM HSCs (HSCs; defined by CD34^+^CD38^−^CD45RA^−^ cells; n = 6) and blasts from patients with AML (n = 198). In total, 29 genes (that encode NTAL and its interactors) were differentially expressed in patients with AML, with the majority (22 genes; 45%) showing upregulated expression in AML samples (*p* < 0.05; Table 2). Finally, using the PRECOG databank (21), which comprehends eight independent transcriptomic studies using patients with AML, we evidenced that 12 of 49 NTAL interactors were significantly associated with OS in patients with AML, in at least two independent public datasets (Fig. 7). Taken together, these findings suggest that NTAL and its interactors may constitute an important group of proteins for AML cell biology, potentially involved in the same pathway associated with tumor growth and stemness characteristics that reflects a subcategory of poor prognostic AML patients.

**Table 2:** Gene expression of NTAL and its interactors in AML patients and healthy HSC—BloodSpot and GSE30029 datasets

Gene name	Description	Log2 expression levels (median, range)		*p* Value
		Healthy HSC	AML blasts	
NTAL	Linker for activation of T-cells family member 2	7.43 (6.71, 8.11)	7.55 (2.14, 10.1)	0.105
LAMTOR5	Ragulator complex protein LAMTOR5	9.2 (8.76, 9.45)	9.84 (6.9, 11.07)	0.006∗∗
LAMTOR1	Ragulator complex protein LAMTOR1	7.85 (7.05, 8.75)	8.5 (5.7, 9.7)	0.051
LAMTOR2	Ragulator complex protein LAMTOR2	6.68 (5.47, 7.4)	8.09 (5.07, 9.76)	0.0042∗∗
LAMTOR3	Ragulator complex protein LAMTOR3	5.42 (4.74, 6.54)	4.34 (2.8, 6.25)	0.0001∗∗∗
LYN	Tyrosine-protein kinase Lyn	10.15 (9.73, 10.51)	10.2 (7.58, 12.24)	0.460
CD44	CD44 antigen	10.3 (8.89, 11.1)	12.1 (8.26, 14.18)	0.003∗∗
HCK	Tyrosine-protein kinase HCK	3.5 (2.69, 3.91)	7.5 (3.76, 10.3)	0.001∗∗∗
INPP5D	Phosphatidylinositol 3,4,5-trisphosphate 5-phosphatase 1	9 (7.99, 9.52)	7.98 (6.3, 12.1)	0.029∗
YES	Tyrosine-protein kinase Yes	8.43 (7.1, 9.82)	6.21 (3.5, 10.18)	0.001∗∗∗
GNG5	Guanine nucleotide-binding protein G(I)/G(S)/G(O) subunit gamma-5	8.5 (8.28, 9.01)	10.28 (7.44, 11.13)	0.001∗∗∗
CHCHD2	Coiled-coil-helix-coiled-coil-helix domain-containing protein 2	9.26 (8.61, 10.13)	10.68 (8.4, 11.1)	0.002∗∗
CD59	CD59 glycoprotein	10.33 (9.72, 10.67)	6.35 (3.15, 10.8)	0.0001∗∗∗
GNG10	Guanine nucleotide-binding protein G(I)/G(S)/G(O) subunit gamma-10	104 (40.2, 291, 6)	69.9 (15.4, 178.1)	0.054
NACA	Nascent polypeptide-associated complex subunit alpha	13.4 (13.28, 13.49)	13.2 (11.18, 13.5)	0.001∗∗∗
RGS19	Regulator of G protein signaling 19	7.47 (7.02, 8.11)	8.53 (4.8, 9.82)	0.0007∗∗∗
RFTN1	Raftlin	5.64 (3.68, 7.01)	5.32 (4.8, 5.77)	0.642
BST2	Bone marrow stromal antigen 2	9.2 (7.62, 9.73)	7.07 (4.23, 9.8)	0.002∗∗
GNG2	Guanine nucleotide-binding protein G(I)/G(S)/G(O) subunit gamma-2	5.16 (2.73, 5.95)	7.87 (6.19, 11.1)	0.001∗∗∗
GNAI3	Guanine nucleotide-binding protein G(k) subunit alpha	8.9 (8.03, 9.8)	9.77 (7.77, 11.3)	0.043∗
PAG1	Phosphoprotein associated with glycosphingolipid-enriched microdomains 1	3.5 (2.93, 6.14)	7.35 (2.5, 10.15)	0.001∗∗∗
CSK	Tyrosine-protein kinase CSK	8.2 (7.64, 8.78)	8.25 (6.19, 9.96)	0.862
COX5B	Cytochrome C Oxidase Subunit 5B	8.77 (8.03, 8.95)	10.05 (6.25, 11.04)	0.0024∗∗
MARCKS	Myristoylated Alanine Rich Protein Kinase C Substrate	4.99 (3.85, 5.51)	8.26 (2.84, 12.22)	0.0085∗∗
RP2	RP2 Activator of ARL3 GTPase	4.62 (4.24, 4.92)	6.001 (3.48, 8.47)	0.0001∗∗∗
SSSCA1	Sjoegren syndrome/scleroderma autoantigen 1	6.35 (5.42, 7.03)	7.12 (4.25, 9.56)	0.071
TESC	Calcineurin B homologous protein 3	4.87 (3.88, 6.32)	7.01 (4.26, 10.4)	0.001∗∗∗
GRB2	Growth factor receptor-bound protein 2	8.5 (7.9, 8.98)	8.85 (6.6, 10.2)	0.072
PHB	Prohibitin	8.26 (7.26, 9.48)	8.12 (4.19, 10.1)	0.359
GNG7	Guanine nucleotide-binding protein G(I)/G(S)/G(O) subunit gamma-7	6.2 (6.02, 7.07)	6.32 (4.4, 8.38)	0.667
LGALS9	Galectin-9	6.5 (6.09, 7.46)	7.25 (5.56, 8.55)	0.029∗
LGALS1	Galectin-1	4.48 (3.8, 5.95)	10.12 (4.7, 12.26)	0.001∗∗∗
ERLIN2	Erlin-2	7.03 (6.28, 8.41)	7.01 (4.17, 8.77)	0.793
FLOT1	Flotillin-1	7.4 (5.92, 8.48)	8.64 (5.47, 10.99)	0.020∗
FLOT2	Flotillin-2	6.75 (5.89, 7.11)	6.95 (5.06, 8.5)	0.170
HPCAL1	Hippocalcin-like protein 1	6.3 (5.91, 6.49)	6.2 (4.03, 7.93)	0.617
PHB2	Prohibitin-2	10.02 (9.53, 10.13)	10.05 (7.89, 10.84)	0.974
GNAI2	Guanine nucleotide-binding protein G(i) subunit alpha-2	8.4 (7.38, 9.34)	10.05 (8.22, 11.7)	0.003∗∗
PRNP	Prion Protein	6.2 (5.35, 7.91)	7.9 (5.33, 9.88)	0.001∗∗∗
NDUFA13	NADH:Ubiquinone Oxidoreductase Subunit A13	7.6 (6.7, 8.46)	9.3 (5.13, 10.58)	0.0054∗∗
TMEM43	Transmembrane Protein 43	8.31 (7.59, 8.92)	8.29 (6.08, 10.6)	0.843
OGDH	Oxoglutarate Dehydrogenase	5.8 (4.52, 6.36)	6.18 (4.29, 8.73)	0.134
GNB1	Guanine nucleotide-binding protein G(I)/G(S)/G(T) subunit beta-1	8.36 (7.58, 9.74)	9.95 (6.6, 11.29)	0.004∗∗
SH3KBP1	SH3 Domain Containing Kinase Binding Protein 1	7.85 (7.1, 8.46)	8.38 (5.24, 9.67)	0.0337∗
TMEM173	Stimulator of interferon genes protein	8.95 (7.06, 9.72)	8.66 (4.88, 10.82)	0.872
PSMD7	Proteasome 26S Subunit, Non-ATPase 7	8.44 (7.66, 8.805)	8.25 (4.64, 9.01)	0.3612
LRRFIP1	LRR Binding FLII Interacting Protein 1	11.49 (11.1, 11.75)	10.73 (8.97, 12.29)	0.0075∗∗
TIMM50	Translocase of Inner Mitochondrial Membrane 50	6.15 (5.43, 6.95)	6.81 (3.76, 9.32)	0.0677
STOML2	Stomatin Like 2	7.44 (7.01, 8.56)	7.98 (4.52, 9.49)	0.2702
NSF	N-Ethylmaleimide Sensitive Factor, Vesicle Fusing ATPase	6.31 (6.12, 6.96)	5.94 (2.45, 7.49)	0.0735

∗ *p* > 0.05, ∗∗ *p* > 0.01, and ∗∗∗ *p* > 0.001.

## Discussion

We have demonstrated that NTAL downregulation is associated with reduced cell viability and proliferation in APL and also leading to decreased tumor burden *in vivo* xenograft models of mantle cell lymphoma and leukemia (9, 30). Here, we further extended our study of NTAL in AML, initially evaluating NTAL gene expression between several neoplastic cell lines using the Cancer Cell Line Encyclopedia. We observed that NTAL is expressed among carcinomas but highly expressed in hematopoietic and lymphoid neoplasms. We also confirmed that in AML cell lines, NTAL–KD impacts on Akt protein phosphorylation and downstream targets. In addition, NTAL–KD led to decreased cell proliferation and decreased tumor mass in murine xenograft models. Using IP–MS strategy in NB4 and U937 leukemia cell lines, we identified 49 potential NTAL protein interactors, including a few previously identified, such as Grb2, Lyn, and SHIP1/INPP5D (24), and new proteins not previously reported as NTAL interactors.

Among the new interactions identified as specifically occurring in the proximity of the membrane or lipid rafts, we highlight LAMTOR1 and CD44. LAMTOR1 is part of the Ragulator complex and directly responsible for anchoring the Ragulator complex to membranes, being involved in the activation of mTORC1. Activated Ragulator and Rag GTPases function as a scaffold recruiting mTORC1 to lysosomes where it is in turn activated. Also, it may regulate the MAPK signaling pathway through the recruitment of some of its components to late endosomes and play a role in RHOA activation. CD44 is a cell surface receptor acting on cell-cell interactions, cell adhesion, and migration, monitoring changes in the tissue microenvironment (31, 32, 33). In T lymphocytes, CD44 participates in hematopoiesis, inflammation, and response to bacterial infection (34). Similar to NTAL, CD44 serves as a platform for signal transduction (35, 36) for PKN2, Rho GTPases RAC1 and RHOA, Rho kinases, and phospholipase C. CD44 is highly expressed in many cells and most abundantly in cells of the hematopoietic system and is also known as a stem cell marker, first described for HSCs and later on confirmed for cancer- and leukemia-initiating cells (37). Interestingly, we had previously observed the association between NTAL and CD44 in another study, since both were downregulated in cells treated with alkylphospholipids (8).

NTAL is tightly regulated by post-translational modifications, which modulate several intracellular pathways, some of them related to PI3K/Akt signaling pathway (8). NTAL is expressed at variable levels by blasts from patients with AML harboring different genetic abnormalities (38). Forced expression of NTAL blocked ATRA- or phorbol ester–induced cell differentiation (38). Several of these interactors have been functionally evaluated regarding the AML cell biology to understand the biological significance described in the clinical setting. When combined, the NTAL interactors predicted survival in AML but not in other hematological malignancies, although our group has demonstrated a functional potential of NTAL protein in mantle cell lymphoma (30). It is tempting to speculate that our signature genes might reflect the activation of AML-specific biological programs, which may impact on patient outcome.

In addition to palmitoylation, which anchors NTAL bound to the intracellular surface of the plasma membrane, several phosphorylation sites have been described as important for its function (2). We identified four members of Src family of protein tyrosine kinases (Lyn, Fyn/Yes, Csk, and Hck), expressed in lymphoid and myeloid hematopoietic lineages (39) that may be potentially involved in the phosphorylation and regulation of NTAL interactions and activity (2). The presence of these proteins strengthens our results and supports the reliability of identification of new interactions, overcoming IP limitations such as lack of specificity.

Here, we evaluated NTAL and its interactors in the context of AML patient expression using a massive amount of information available in public databases. We found that most NTAL interactors are upregulated in a large subset of patients with AML. More importantly, NTAL and its interactors were associated with an GMP-like molecular signature, showing a more oxidative phosphorylation–dependent metabolic signature, which was further validated when we compared the levels of NTAL and its interactors in AML samples with an immunophenotype profile more L-GMP *versus* patients with a more L-lymphoid-primed multipotent progenitor/L-HSC. This suggests that patients with AML that express high levels of NTAL and its interactors differ significantly in their origin, metabolic state, and cell cycle state, compared with patients with low NTAL interactor expression (40). Also, patients with high expression of NTAL protein and their interactors presented a positive correlation with the genes of the *HOXA*, *HOXB*, *RAS*, and *AKT1* family, also enriched in particular leukemic subtypes, such as NPM1 mut/DNMT3A mut AMLs (also observed in our GSEA) (41). Interestingly, recent studies (42, 43, 44) have shown that this subtype of AML can be targetable by the use of menin–mixed lineage leukemia) inhibitors. Since our data showed that patients with high NTAL (and its interactors) expression exhibited similar genetic signature to this group of patients, it is conceivable that the treatment with menin–mixed lineage leukemia inhibitors may also work in this group of patients, even in the absence of NPM1/DNMT3A mutation. Particularly, the gene expression correlation in patients among our molecular signature and AKT1 increases the pieces of evidence of the important participation of NTAL in Akt pathway (8). Finally, patients with low expression of NTAL and its interactors show enrichment of process associated with cell cycle arrest, which confirms our results upon NTAL KD in AML cell lines.

In conclusion, we demonstrated that NTAL is an important lipid raft protein in leukemia. NTAL mediates protein–protein interactions and signal transduction associated with a GMP-like signature. These molecular interactions potentially affect the metabolic state and cell cycle state through a signalosome complex and can culminate in potential targets to selectively treat aggressive leukemia with a progenitor-like cell pattern.

## Data availability

The MS proteomics data have been deposited to the ProteomeXchange Consortium *via* the PRIDE (45) partner repository with the dataset identifier PXD005850.

## Supplemental data

This article contains supplemental data.

## Conflict of interest

The authors declare no competing interests.
