# Supplementary material for: The Expression of NTAL and Its Protein Interactors Is Associated With Clinical Outcomes in Acute Myeloid Leukemia
Source: Mol Cell Proteomics. 2021 May 7;20:100091. doi: 10.1016/j.mcpro.2021.100091 (PMC8220000; doi:10.1016/j.mcpro.2021.100091)
Supplement: Supplemental Table S2 and Figure S1 [file mmc2.docx]

**NTAL network of protein interactions is associated with clinical outcomes in acute myeloid leukemia.**

Carolina Hassibe Thomé^1,2^*, Germano Aguiar Ferreira^1,2*^, Diego Antonio Pereira-Martins^2,3*^, Guilherme Augusto dos Santos^2^, Douglas R. Almeida-Silveira^,~~4,~~5^, Isabel Weinhäuser^2,3^, Gustavo Antônio de Souza^6^, Roos Houtsma^3^, Jan Jacob Schuringa^3^, Eduardo M. Rego^2,4#^ and Vitor M. Faça^1,2#ϯ^

**ϯ Corresponding authors:** Vitor M. Faça, Ribeirão Preto Medical School, Department of Biochemistry and Immunology and Center for Cell Based Therapy, University of Sao Paulo. Av. Bandeirantes, 3900, Ribeirão Preto, SP 14048-900, Brazil.

Email: [vitor.faca@fmrp.usp.br](mailto:vitor.faca@fmrp.usp.br)

Supplementary Table 1: Sources and properties of antibodies used in the work.

|  | **Antibody** | **Company** | **Catalog**  **Number** | **Source** | **P/M** | **Mass**  **(kDa)** | **Application** | **Dilution** |
| --- | --- | --- | --- | --- | --- | --- | --- | --- |
| 1 | β-actin | Sta Cruz Biotechnology | sc-81178 | Mouse | M | 43 | WB | 1:500 |
| 2 | CD44 | Cell signaling | #3570 | Mouse | M | 80 | IF,  WB | 1:500,  1:1000 |
| 3 | Fibrillarin | Cell signaling | #2639 | Rabbit | M | 37 | WB | 1:1000 |
| 4 | Hck | Cell signaling | #14643 | Rabbit | M | 59, 61 | IP,  IF,  WB | 1:50,  1:50,  1:1000 |
| 5 | IgG XP^®^ Isotype Control | Cell signaling | #3900 | Rabbit | M | - | IP | 1:50 |
| 6 | Lamtor1 | Cell signaling | #8975 | Rabbit | M | 18 | WB,  IP,  IF | 1:1000, 1:50,  1:50 |
| 7 | Lamtor5 | Cell Signaling | #14633 | Rabbit | M | 10.5 | WB,  IP,  IF | 1:1000, 1:50,  1:50 |
| 8 | LAMP1 | Cell signaling | #9091 | Rabbit | M | 120 | WB | 1:1000 |
| 9 | Lyn | Cell signaling | #2732 | Rabbit | P | 56 | IP,  WB | 1:50,  1:1000 |
| 10 | NTAL | Cell signaling | #9533 | Rabbit | P | 25 | WB,  IP | 1:1000, 1:50 |
| 11 | NTAL Biotinylated | R&D Systems | BAF4066 | Sheep | P | 25 | WB | 1:250 |
| 12 | NTAL | Thermo Scientific | NAP-70 | Mouse | M | 30 | IF | 1:50 |
| 13 | SHIP1 | Cell signaling | #2727 | Rabbit | M | 145 | IP,  IF,  WB | 1:50,  1:100,  1:1000 |
| 14 | β-tubulin | Cell signaling | #2146 | Rabbit | P | 55 | WB | 1:1000 |
| 15 | Yes/FYN | Cell signaling | #3201 | Rabbit | P | 60 | IP,  WB | 1:50,  1:1000 |
| 16 | p44/42 MAPK (Erk1/2) | Cell signaling | #4695 | Rabbit | M | 42, 44 | WB | 1:1000 |
| 17 | phospho-p44/42 MAPK (Erk1/2) (Thr-202/Tyr-204) Biotinylated | Cell signaling | #4094 | Rabbit | M | 42,44 | WB | 1:1000 |
| 18 | AKT | Cell signaling | #9272 | Rabbit | P | 60 | WB | 1:1000 |
| 19 | phosphor-AKT (Ser473) | Cell signaling | #4058 | Rabbit | M | 60 | WB | 1:1000 |
| 20 | Ras | Cell signaling | #3339 | Rabbit | M | 21 | WB | 1:1000 |
| 21 | Caspase-3 | Cell signaling | #9662 | Rabbit | P | 17, 19, 35 | WB | 1:1000 |
| 22 | NTAL | Sigma | HPA003462 | Rabbit | P | 26.6 | IHC | 1:100 |

* P = Polyclonal; M = Monoclonal; WB = Western Blotting, IP = Immunoprecipitation, IF = Immunofluorescence, IHC= Immunohistochemistry

Supplementary Table 2: Identification of NTAL protein interactors by IP-MS/MS.


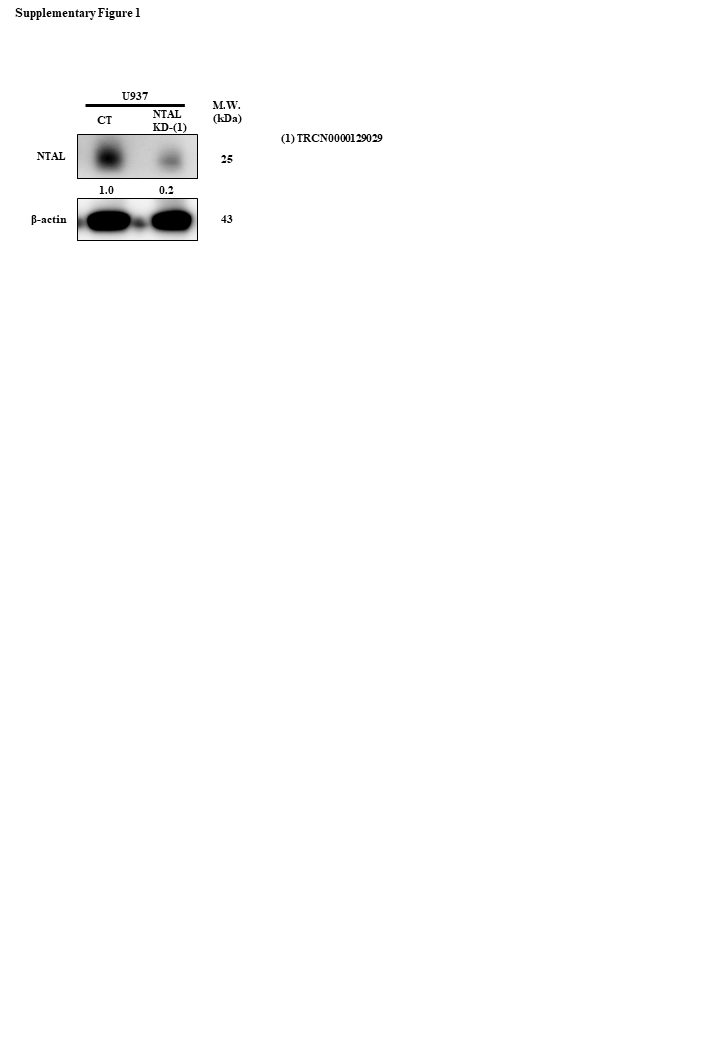


**Supplementary Figure 1:** NTAL knockdown was obtained from U937 cells via lentiviral transduction of shRNA that targets NTAL.
